# Non-Specific Responsive Nanogels and Plasmonics to Design MathMaterial Sensing Interfaces: The Case of a Solvent Sensor

**DOI:** 10.3390/s222410006

**Published:** 2022-12-19

**Authors:** Nunzio Cennamo, Francesco Arcadio, Fiore Capasso, Devid Maniglio, Luigi Zeni, Alessandra Maria Bossi

**Affiliations:** 1 Department of Engineering, University of Campania Luigi Vanvitelli, Via Roma 29, 81031 Aversa, Italy; 2 Department of Industrial Engineering, BIOtech Research Center, University of Trento, Via delle Regole 101, Mattarello, 38123 Trento, Italy; 3 Department of Biotechnology, University of Verona, Strada Le Grazie 15, 37134 Verona, Italy

**Keywords:** responsive nanomaterials, plasmonics, chemical sensor, MathMaterial, artificial intelligence, interface, nanogel, multidimensional sensing

## Abstract

The combination of non-specific deformable nanogels and plasmonic optical probes provides an innovative solution for specific sensing using a generalistic recognition layer. Soft polyacrylamide nanogels that lack specific selectivity but are characterized by responsive behavior, i.e., shrinking and swelling dependent on the surrounding environment, were grafted to a gold plasmonic D-shaped plastic optical fiber (POF) probe. The nanogel–POF cyclically challenged with water or alcoholic solutions optically reported the reversible solvent-to-phase transitions of the nanomaterial, embodying a primary optical switch. Additionally, the non-specific nanogel–POF interface exhibited more degrees of freedom through which specific sensing was enabled. The real-time monitoring of the refractive index variations due to the time-related volume-to-phase transition effects of the nanogels enabled us to determine the environment’s characteristics and broadly classify solvents. Hence the nanogel–POF interface was a descriptor of mathematical functions for substance identification and classification processes. These results epitomize the concept of responsive non-specific nanomaterials to perform a multiparametric description of the environment, offering a specific set of features for the processing stage and particularly suitable for machine and deep learning. Thus, soft MathMaterial interfaces provide the ground to devise devices suitable for the next generation of smart intelligent sensing processes.

## 1. Introduction

Bio/chemical sensing is a two-component process in which a recognition element and a transducer jointly work to generate a measurable response in the presence of the target analyte. Recognition elements, that span across a variety of materials, such as inorganics, organics, and biologicals, are meant to interact with the analyte molecule and are contiguous to the transducer. Transducers, meant to translate a molecular interaction into a measurable electronic signal, belong to different classes depending on their physical working principles (electrochemical, piezoelectric, electrical, electronic, optical, etc.) [1].

In a typical sensor’s design, the chosen recognition element should match the transducer [2] and ensure a specific and selective interaction with the analyte. It follows the paradigm that a one-sensor system provides specific information about a single analyte. Under this model, multi-sensorial functions can be achieved by the sum of several one-sensor units. Clustering together, sensors provide multiple-sensing platforms, i.e., sensor arrays. Arrays consist of different recognition elements; a similar transducer, multiple transducers, or both [3]. The sensor array provides a multi-faced description of the system under analysis by combining the outputs of each single element. The epitome of the one-sensing unit’s array is the electronic nose (E-nose) [4]. The E-nose is a multi-sensorial device whose outputs are response patterns, which allows the identification, comparison, and quantitation of complex systems, including data storage and retrieval. In the E-nose, the identification of the analytes composing a complex mix is actuated by recognition patterns. For this, each single sensor in the array is intended to have the narrowest specificity and is designed to be unique in the device. Within such a model, we witness technological and scientific advances to improve the specialization of single-sensor units.

In the present work, we aim to overturn such a paradigm by performing multi-sensing through a generalistic recognition layer. We propose a sensor that relies on a quasi-non-specific material with more than one freedom degree and thus can undergo more physico-chemical changes in response to the environment. The non-specific material is grafted onto the transducer. The expected readout signals are sets of changes occurring to the non-specific material in reply to different environments, thus resulting in specific patterns of signals. Hence, the non-specific material, layered onto the transducer and coupled to data processing, turns into a MathMaterial Sensor. Moreover, such an approach epitomizes the paradigm swap: a non-specific surface for specific sensing.

The shift from ultra-specialized to non-specialized is taking place in many areas, albeit such a tendency and its potential are still rather underestimated. Examples of this cultural transition are in cell sorting utilizing image pattern analysis [5] or using back-scattering analysis for sorting nanoparticles by training artificial intelligence [6]. Concerning sensing, the key condition for moving to the non-specialized approach is to choose a quasi-general material with smart properties, such as responsivity, strain, and structural transition [7]. Smart materials undergo more than one physico-chemical change, induced by and dependent on the environment. It follows that layering a smart material on a transducer would enable one to gain information about more than a single characteristic of the environment, embodying the concept of “general recognition for specific sensing”.

In this context, organic polymers are ideal materials for mapping complex systems [8]. Particularly suited to the task are hydrogels, three-dimensional polymeric materials characterized by a high water content [9]. The hydrogel’s polymer network is flexible and changes shape in response to stimuli (e.g., pH, temperature, solvent). Swelling and shrinking of the hydrogel occur when the surrounding environment changes [10]. The charge density, the type of pendant chains, and the degree of cross-linking of a hydrogel are generally capable of influencing the time and the extent of the network response to the applied stimulus. Hence, by playing with the hydrogel composition, a wide degree of responses can be obtained.

Indeed, flexible hydrogels have been exploited for optoelectronic devices [11,12]. Sensors based on hydrogel-made optical fibers, bearing flexible and stretchable properties, have been proposed [13]. Thermo- and solvent-responsive hydrogels have been demonstrated to provide improved optical sensitivity [14]. Substantial enhancement in sensitivity has also been achieved using soft molecularly imprinted nanogels if/when coupled to plasmonics [15,16,17].

In the present work, the “non-specific material for specific sensing” consisted of soft nanogels grafted to a D-shaped POF plasmonic probe [18]. The POF was chosen for its versatility of configurations, ease of manipulation, low cost, great numerical aperture, large diameter, use of white light sources, and the possibility of remote interrogation [19]. The nanogel–POF was placed in different environments (i.e., solvents). It was anticipated that the optical mapping of the deformations occurring to the nanogels would provide a multiparametric description of the surrounding environment, matching the demands for versatile and easy-to-produce devices that fully satisfy the demands of sensing approaches based on machine and deep learning.

## 2. Materials and Methods

### 2.1. Chemicals

Acrylamide (Aam), (R)-(+)-α-lipoic acid, Ammonium persulfate (APS), 1-Ethyl-3-(3-dimethylaminopropyl)carbodiimide hydrochloride (EDC), Glycerol, Lysil-lysine (Lys-Lys), Methacrylic acid (MAA), N,N′-methylene bisacrylamide (BIS), N-hydroxysuccinimide (NHS), N-*tert*-butylacrylamide (TBAm), 2-propanol, Sodium dihydrogen phosphate monohydrate (NaH2PO4∙H2O), Disodium hydrogen phosphate dehydrate (Na_2_HPO_4_∙_2_H_2_O), N,N,N′,N′-tetramethyl ethylenediamine (TEMED), were from Sigma-Aldrich (Darmstadt, Germany). A 40% *w*/*w* APS stock solution was freshly prepared prior to use.

### 2.2. Synthesis of Nanogels

Monomer’s stock solutions were prepared in MilliQ water at 2% (*w*/*v*); TBAm stock solution was prepared in ethanol. The total monomer concentration was 0.2% (*w*/*v*). The final volume was 10 mL. The polymerization (details in Supplementary Materials Section S1) was prepared by mixing Aam:MAA:TBAm:BIS at the mol percentages of 8, 8, 4, and 80%, respectively, with MilliQ water and sonicated for 10 min. Solutions were filtered on a 0.2 µm cellulose filter (Sartorius Stedim, Firenze, Italy). Vials were closed with rubber caps and bubbled with N_2_ for 10 min. Polymerization was initiated by TEMED (0.3 µL/mL) and APS (1 µL/mL) at room temperature for 20 h at 20 °C. The polymerization yield was calculated from the dry weight of the nanogels, after lyophilization, with respect to the total weight of the monomers added to the synthetic batch.

### 2.3. Dynamic Light Scattering

Size distribution and the polydispersity index (PDI) were determined using a Zetasizer Nano ZEN3600 (Malvern Instruments Ltd., Worcestershire, UK) equipped with a 633 nm He-Ne laser. Nanogels were dispersed in filtered deionized water at 1 mg/mL and filtered at 0.22 µm prior to measurement. The material refractive index (RI) was 1.490 and the absorption value 0.01; the dispersant RI was 1.332 for water and 1.340 for PBS, the viscosity was 0.89 cP for water and 1.02 cP for PBS as reported by Zetasizer v.6.32 software (Malvern Instruments Ltd., Worcestershire, UK). The temperature was set at 298 K, and a detection angle of 173° was used. Measurements were in triplicate.

### 2.4. Measurement of the Swelling Ratio

The swelling ratio (SR) of the nanogels (V/Vo) was determined at DLS using the Zetasizer Nano ZEN3600, detailed in Section 2.3. SR was measured from the ratio of their diameters swollen in water (d_o_) and ethanol/water mixtures (d), according to the equation SR = (d/d_o_)^3^, as explained in [20].

### 2.5. SPR POF Platform

The POF platforms of PMMA and 1 mm diameter were prepared by removing the cladding from the POF along half the circumference, spin coating the exposed core at 6000 rpm for 60 s with a buffer layer of photoresist Microposit S1813 (MicroChem Corp., Westborough, MA, USA) to a final thickness of about 1.5 μm, and finally sputtering a thin gold film (three times with a current of 60 mA for 35 s, 20 nm per step, and a total thickness of 60 nm) using a sputtering machine (Bal-Tec SCD 500), as reported in [18]. The D-shaped sensing region was about 10 mm in length. The RI values of the materials, in the visible range of interest, are 1.49 for PMMA, 1.41 for fluorinated polymer, and 1.61 for Microposit S1813 photoresist. Typically, the SPR-POF platform has reported bulk sensitivity equal to about 2000 nm/RIU, a resolution around 10^−4^ RIU, and a figure of merit (FOM) equal to about 17 RIU^−1^ [18]. It is worth noting that the RI measured by the SPR platform (e.g., the SPR-POF probe) depends on the external temperature (the temperature could change the RI of the liquid in contact with the SPR sensing surface). Moreover, when dealing with alcoholic solutions, evaporation can also cause a variation in the measured RI value. In this work, these effects were mitigated since the measurements were carried out at a constant room temperature (about 20 °C), by using short incubation times, and by checking the refractive index of the solutions using an Abbe refractometer (RMI, Exacta Optech, Wolfratshausen, Germany). However, both these effects can be eliminated by utilizing thermostabilized cells.

### 2.6. Preparation of the Nanogel–POF Platform

Nanogels were coupled to the POF, as described in [16]. A SAM was formed by incubating the gold POF surface in a 300 µM (R)-(+)-α-lipoic acid in ethanol 8% *v*/*v* solution overnight. After rinsing with water and drying, the POF was incubated for 1 h in 10 mM Lys-Lys, and 50 mM EDC/NHS (1:1 mol:mol) in MES buffer 10 mM pH 5.5. Nanogels (1 mg/mL) were re-suspended in an MES buffer for 3 h under mild agitation and filtered on a 0.22 µm filter, and 40 mM EDC and 10 mM NHS were added. 200 µL were placed onto the POF and allowed to react for 2 h at room temperature in a sealed humid box. Nanogel–POFs were then washed extensively in 15 mL Falcon tubes with water prior to use.

### 2.7. Analysis of the Nanogel–POF by Atomic Force Microscopy

Surface topography was studied using an NT-MDT Solver Pro system equipped with an S7 scanner. All samples were imaged in semi-contact mode using silicon tips (NSG-11, NT-MDT, 10 nm nominal tip radius, resonance frequency of 181 kHz), collecting 1 × 1 µm, 512 points resolution images, acquired on different regions of each sample. AFM data were analyzed with the support of Gwyddion analysis software 2.2. Topography and phase contrast data were collected.

### 2.8. Preparation of the Solutions for Test-Measurements

The glycerol:water and isopropanol:water mixtures were prepared as reported in [21] and [22], respectively. More specifically, the 10% (*v*/*v*) and 17% (*v*/*v*) glycerol solutions and the 10% (*v*/*v*) and 20% (*v*/*v*) 2-propanol solutions were prepared in MilliQ water. Prior to testing, the RI of each solution was measured with the Abbe refractometer.

### 2.9. MathMaterial Sensor Measurements

The nanogel–POF chip was placed in a sealed cell suitable for sample introduction. Measurements were carried out by placing a volume of a solution of 50 μL on the sensing area. The optical signal was measured over 10 min, at time intervals of 1 min, starting from the sample application time (t = 0). The measurements were performed at room temperature in triplicate. The minimum of the optical signal was then plotted as a function of the RI of the tested solutions and as a function of time.

## 3. Results and Discussion

### 3.1. Responsive Behaviour of the Soft Nanogels in Solvent

**Figure 1:**
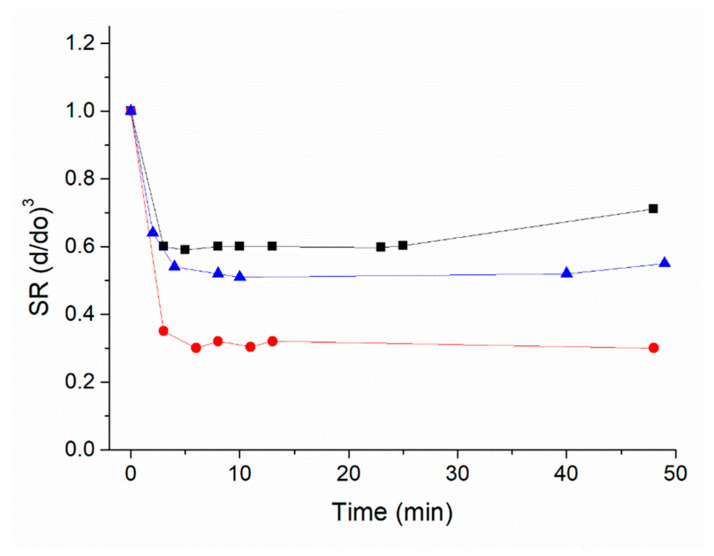
Flexibility of the nanogels monitored as a modification of the hydrodynamic size with the addition of isopropanol to the water suspension. Blue track: isopropanol:water 1:99 *v*/*v*; Black track: isopropanol:water 1:19 *v*/*v*; Red track: isopropanol:water 3:17 *v*/*v*. Measurements were in triplicate; StDv < 10%.

Nanogels were synthesized from a combination of acrylamide-monomers (acrylamide, *tert*-butylacrylamide), methacrylic acid, and N,N′-methylene bisacrylamide as a reticulating agent, resulting in homogeneous nanomaterials with a Z-average of 89.5 ± 0.5 and a polydispersity index (PDI) of 0.18 (details in Supplementary Materials Sections S1 and S2; Table S2. Zeta potential and Z-average of the nanogels and Figures S1 and S2), similar to what is reported in [23]. Nanogels in water suspension were tested for solvent responsivity by monitoring the shrinking over time (≤30 min) when a low-viscosity solvent was added. Figure 1 shows the deformation of the nanogels in the isopropanol:water mixtures, expressed as a swelling ratio (SR) where SR = V/V_o_ = (d/d_o_)^3^, d being the diameter of the nanogel in the solution and d_o_ the diameter in pure water [20]. Results showed that nanogels shrank to SR ~0.3 of the initial hydrated nanogel’s volume when placed in the isopropanol environment, confirming significant deformability.

### 3.2. Responsive Behaviour of the Soft Nanogel–POF Interface

**Figure 2:**
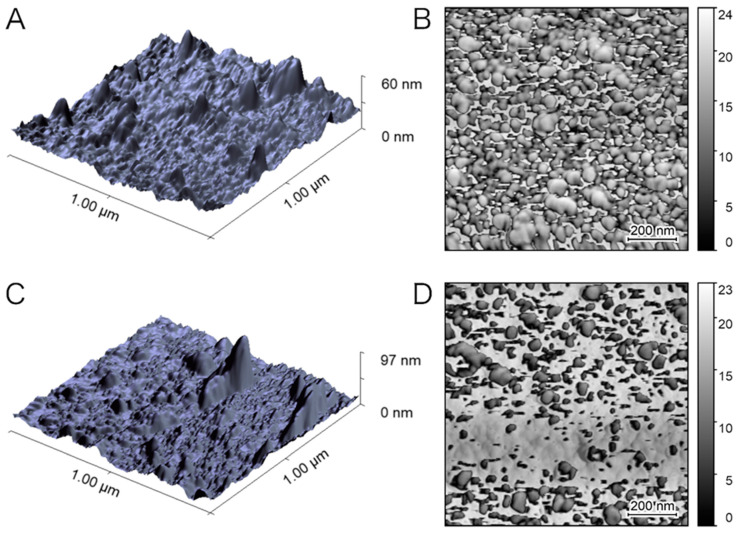
The nanogel–POF interface in wet and dry conditions. Atomic force microscopy was used to investigate: (**A**) surface topography of nanogel–POF probe in a hydrated state, and correlated to its phase (**B**); (**C**) surface topography of dehydrating nanogels on the POF, and correlated to the phase (**D**).

The nanogels were grafted to the D-shaped POF plasmonic probe following the protocol reported earlier [15]. Figure 2 reports the topography and phase contrast images of the nanogel–POF interface, acquired by atomic force microscopy (AFM) [24], for hydrated nanogels on the probe (Figure 2A,B) and for the same nanogels on the probe allowed to dehydrate for 20 min in an airstream (Figure 2C,D). The comparison between the nanogel’s interfaces phases (Figure 2B versus Figure 2D) supported the evidence of swollen (higher phase-lag) and dehydrated (lower phase-lag) hydrogels, referring either to higher energy-dissipating or conservative nanogels.

**Figure 3:**
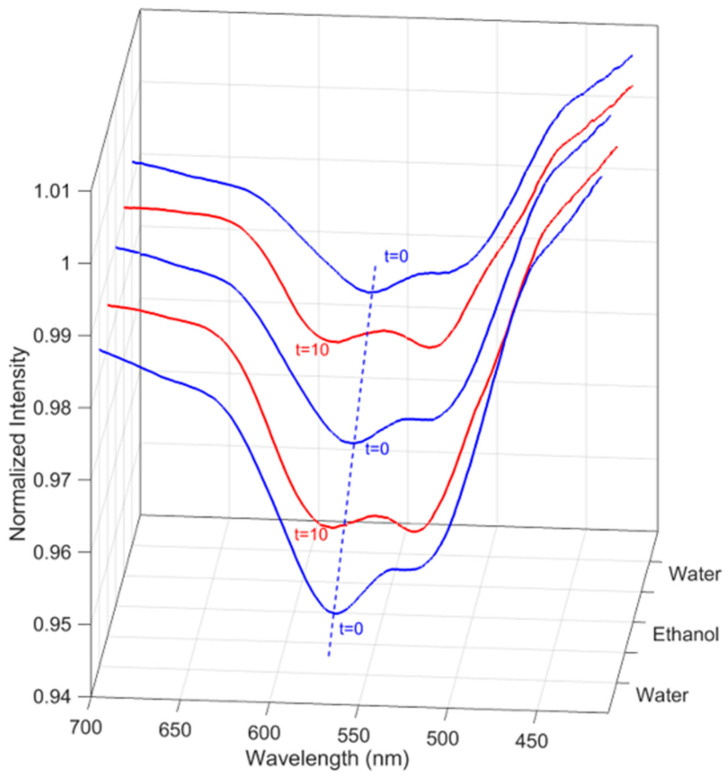
The nanogel–POF interface behaves as a reversible optical switch. The sensor was tested with the following cycle: water was placed on the interface (start of the cycle; t = 0, blue lines), followed by ethanol:water RI 1.339 (10% *w*/*v*), let incubate for 10 min, and then measured (t = 10, red lines). At the cycle’s completion, the sensor was placed in water, incubated for 10 min, and the next cycle was started (t = 0, blue line). Three out of five consecutive cycles performed on the sensor are reported. It can be observed that when alternatively placed in water (blue line) and ethanol:water RI = 1.338 (red line), the optical minima shift between typical positions, hinting at possible uses of the nano-gel-POF as an optical switch.

Next, the nanogel–POF interface was tested by exploiting the plasmonic phenomenon via the experimental setup reported in Supplementary Materials Section S4 (Figure S4: Image of the device). The nanogel–POF interface was exposed to repeated water cycles as the surrounding environment, followed by ethanol:water RI = 1.339 (tested cycles *n* = 5), and the optical information, expressed as λ_min_, was monitored, as reported in Figure 3. The results showed a consistent pattern of Δλ shifts, indicating the reversibility of the volume-to-phase transition processes at the nanogel–POF interface, ultimately resembling an optical switch [8]. Indeed, the on/off mechanism offered by the nanogel deformation might find uses in information storage or delivery in a system that can be described as a polymeric switch [25].

### 3.3. Towards MathMaterial Sensing

**Figure 4:**
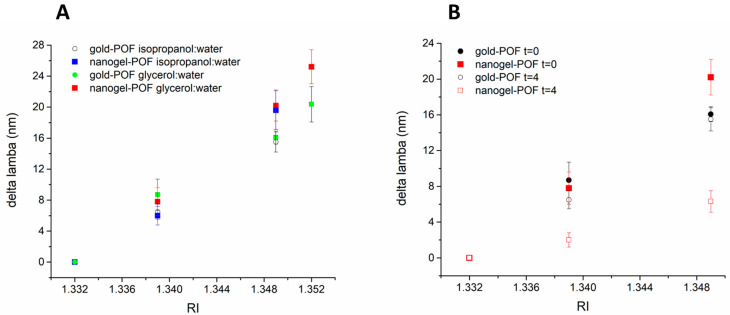
(**A**) The shift of the plasmonic minimum (Δλ) is plotted as a function of the refractive index (RI) of the surrounding environment at the initial sample-dropping time (t = 0) for the bare gold POF and nanogel–POFs. For all reported conditions, the Δλ linearly correlated to the RI. (**B**) The Δλ is plotted as a function of the RI of the surrounding environment (water and water:isopropanol mixes) both at the initial sample-dropping time (t = 0) and after 4 min (t = 4) for both bare gold POF and nanogel–POFs. The comparison between incubation times t = 0 and t = 4 min shows that the bare gold POF has an optical response uniquely related to the RI of the surrounding environment. In contrast, for the nanogel–POF, the isopropanol:water mixes that have a documented shrinking effect on the nanogels [27] produced a significant drop in the optical responses (t = 0 red solid squares; t = 4 red open squares). The result highlights optical effects associated with the softness of the nanogels on the probe, hence the possibility to sense the different environments by exploiting soft non-specific materials and monitoring their over-time deformations.

The optical responses of nanogel–POF were tested for different environments. We reported earlier that dry and hydrated nanogels on the POF probe display two distinctive plasmonic minima [15] associated with the modified interaction of the light with the nanogel layer in the two extreme hydration states, collapsed or swollen. Here, the plasmonic response of the hydrated nanogels on the probe (*n* = 3) was studied for different surrounding media. As a reference, a bare gold POF probe (*n* = 3) was used. The nanogel–POF and bare gold POF platforms were tested with water (refractive index, RI = 1.332) and in surrounding media composed of water/glycerol mixtures (RI = 1.342; RI = 1.350; RI = 1.352). The optical minima measured for each condition were plotted as a function of RI (Figure 4A, green circles; red squares). Additionally, the POF platforms were tested with water/isopropanol mixtures (RI = 1.339; RI = 1.349; RI = 1.350), and the changes in the optical minima were monitored (Figure 4A, open circles; blue squares).

As expected, the Δλ_min_ linearly correlated to the increment in RI of the surrounding medium [26]. As shown in Figure 4A, the shifts in the optical minima at the solvent deposition time (Δλ@t = 0), plotted as a function of the RI of the solvent mix, confirmed the predicted linear correlation between the Δλ and the surrounding medium’s RI in a range of RI encompassing 1.331 to 1.352 both for the bare gold and the nanogel–POFs.

Next, the effect of each surrounding medium was monitored, starting from the initial deposition time (t = 0) to t = 4 min. Both the bare gold POFs and nanogel–POFs were tested (Figure 4B). In the case of the gold POFs, the linear correlation between the RIs of the tested solutions (glycerol:water; isopropanol:water) and the Δλ was confirmed both at t = 0 and at t = 4 min. The bare gold POF enabled the plasmonic assessment of the RIs of the surrounding medium, quasi-irrespectively of the time the interface stays in contact with the solution, albeit evaporation phenomena occur at longer times. Thus, the gold POF can act as an RI sensor.

In contrast, the nanogel–POF’s interface behaved with higher complexity than the bare gold POF, showing more freedom degrees (Figure 4B). Primarily, for measurements at t = 0, the nanogel–POF again acts as an RI sensor. Interestingly, the optical behavior Δλ@t = 4 min indicated that when the surrounding medium is composed of low-density solvents that induce phase-to-volume transitions of the nanogels [27], such as an isopropanol:water mix (Figure 4B, comparison between red solid versus red open squares), the optical information underwent a significant deviation over time. The optical shift over time was hypothesized to result from the nanogel shrinking close to the plasmon wave.

**Figure 5:**
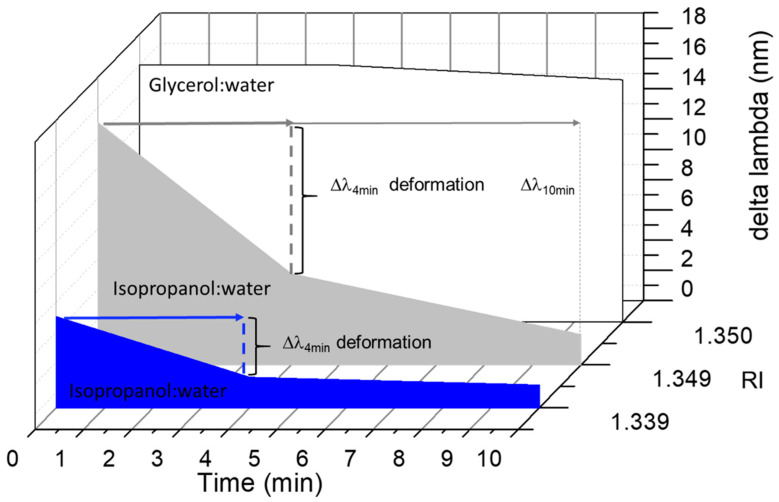
The MathMaterial sensor. Nanogel–POF was placed in contact with different environments: glycerol:water RI 1.350 (17% *v*/*v* glycerol) white; isopropanol:water RI 1.349 (20% *v*/*v* isopropanol) grey; isopropanol:water RI 1.339 (10% *v*/*v* isopropanol) blue. At time t = 0, the optical readouts (Δλ shift) informed us about the RI of the surrounding environments. Extending the measurements to longer incubation times (t = 4) permits monitoring deformations of the nanogel in terms of further Δλ shifts. In the example, glycerol:water had no collapsing effects on the nanogel. The signal (λ) was stable, whereas shrinking environments resulted in Δλ, enabling us to distinguish the nature of the surrounding solvent. Additionally, multiple sampling times (t = 0, t = 4, t = 10) enabled monitoring of the extent of the deformation of the nanogel, gaining kinetics information on the deformation process, and, hence, on the concentration of the solvent.

Thus, the nanogel–POF can be used as a sensory system in which the optical response is modulated by more than a single characteristic of the surrounding medium, i.e., the RI and the shrinking effect. The nanogel–POF allows a two-dimensional measure of the analyte solution, offering the possibility to report on complex environments. As a confirmation, the nanogel–POF was tested in different environments, either suitable to induce shrinking (isopropanol:water) or solutions not altering the 3D nanogel structure (e.g., glycerol:water), and each was monitored [27]. Figure 5 shows that at time t = 0, the optical readouts (Δλ shifts; details in Supplementary Materials Section S3, Figure S3) correlated to the RIs of the surrounding environments, in accordance with the previous data (Figure 4A). Thus, the first level of information accessed using the nanogel–POF system was the RI of the surrounding solution. For the nanogel–POF incubated for longer times in a surrounding environment, the possible extra-deformations of the nanogel structure report on the chemical nature of the surrounding media. These additional volume-to-phase transitions are perceived as further time-dependent Δλ shifts. As a result, the second level of information gained by this non-specific nanogel–POF is about the chemical nature of surrounding environments, enabling us to discriminate shrinkage from swelling compositions.

It is interesting to note that the extent of the deformation induced by the surrounding medium’s physico-chemical characteristics can be modulated by playing with the contact time between the nanogel–POF and the medium (Figure 5). Thus, introducing the time parameter for the measurements (t = 0; t = 4; t = 10) enabled a third level of information on the kinetics of the nanogel collapse, correlated to the concentration of the chemical solvents in the surrounding environment, as demonstrated by the solutions with high isopropanol (RI 1.349) content respective to the lower ones (RI 1.339).

**Figure 6:**
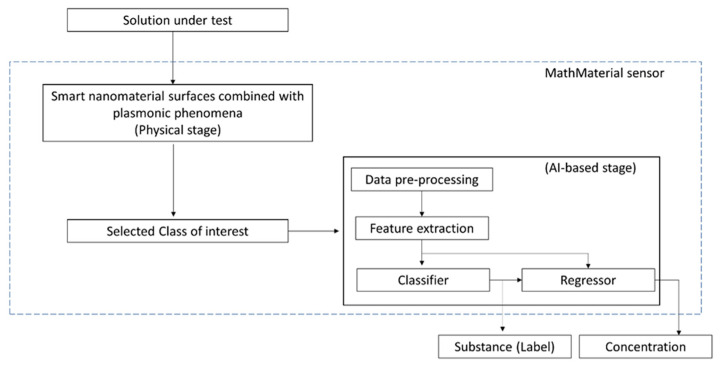
Outline of the MathMaterial sensing approach.

It is expected that by taking advantage of machine learning-based algorithms [28,29], the obtained MathMaterial sensor response can be used to identify a substance (in a determined class) and its concentration using simple data processing, as shown in Figure 6.

To test the concept of MathMaterial machine learning sensing, the MathMaterial sensor was challenged with the simplest scenario, consisting of an ON/OFF response only, detecting whether or not the solution contained alcohol. The physical deformations, ensuing the contact of the smart MathMaterial interface with glycerol:water, or isopropanol:water, were used to feed the algorithm. The MathMaterial sensor is trained to distinguish between the two classes of solutions. The standard MATLAB^®^ toolbox, “Statistics and Machine Learning”, was used to build a prediction model, providing a classification learner able to train models and classify data. By using a set of input data (RIs, yes/no alcohol) and known outputs from the experimental observations (Δλs), supervised machine learning was performed. Once the model was trained, it provided predictive forecasts on the response expected to unknown solutions.

**Figure 7:**
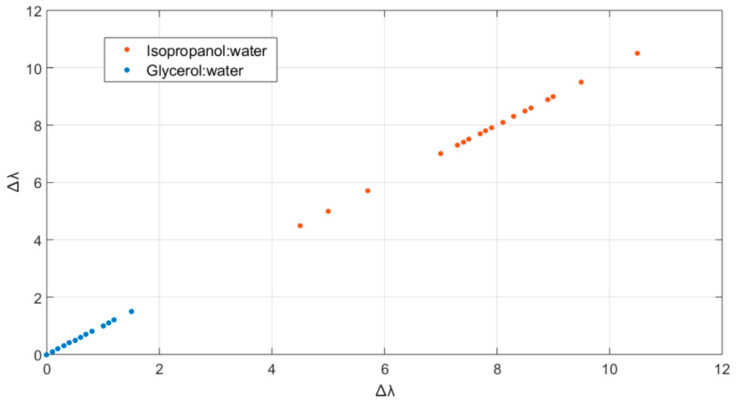
Scatter plot of the implemented dataset: two solutions with the same RI, but with (red, isopropanol:water) and without (blue, glycerol:water) the alcoholic component.

A proper dataset was acquired by testing the nanogel–POF sensors with two solutions, having the fixed and same RI = 1.349 while significantly differing in compositions (glycerol:water, or isopropanol:water). Consistent with the experimental data reported in Figure 5, the Δλ shifts for each solution were acquired after 4 min incubation. The procedure was repeated for *n* = 20 acquisitions for each solution to obtain a robust dataset to train and validate the prediction model. Figure 7 reports the scatter plot showing the dataset clustering. The overlapping of multiple measurements of the same class of solution accounted for the high reproducibility of the MathMaterial sensor response.

**Figure 8:**
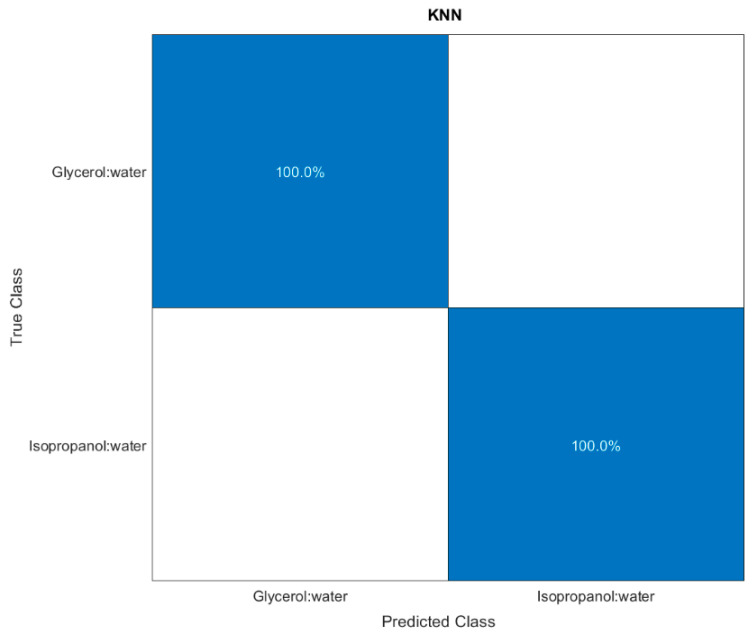
Confusion matrices obtained for the predictions over a stratified cross-validation procedure.

Next, the classification algorithms available on the MATLAB^®^ toolbox were trained. From among all, the K-Nearest Neighbour (KNN) algorithm was chosen for its highest accuracy (100%), as shown by the confusion matrix reported in Figure 8.

The model obtained from the trained algorithm was exploited to test unknown solutions, having a fixed RI equal to 1.349, and by providing the Δλ shifts acquired after 4 min as input data. The prediction demonstrated 100% accuracy, are supported by the well-defined cluster regions of Figure 7.

The herein reported results just concerned a simplified ON/OFF procedure. It is foreseen that including the incubation times in the model (Figure 5) as an additive variable of the dataset will scale up the system’s complexity, hence permitting us to also determine the alcoholic percentage in the solution. It should be noted that expanding the dataset dimensionality leads to the necessity of a larger quantity of input data and longer training times.

## 4. Conclusions

Here, we explored for the first time the effect of coupling nanogels to a POF plasmonic probe as a modification aspect of a state-of-the-art coupling of SPR POF probes with molecularly imprinted nanoparticles (nanoMIPs), synthetic polymeric receptors optimized for recognizing a target analyte using template-assisted synthesis and [15,16,17].

Here, a non-specific but responsive nanogel layer grafted to a POF plasmonic probe empowered the sensor, thanks to its multi-characteristics, ultimately enabling multidimensional measurements. Recalling the concept of MathMaterial, which refers to metamaterials that can be designed to acquire the properties needed for whatever context they will be needed in, our results define the MathMaterial sensors. The MathMaterial sensing is a two-stage process, in which the first “physical stage” occurs via the nanomaterial/plasmonic interface and selects the class of the substance of interest, similar to an ON/OFF approach, whereas the second stage is essentially a classifier, such as an AI-based process, that enables detection of the specific substance from the selected class and eventually its concentration. Thus, the nanogel–POF coupled with AI provides advancement in E-tongue sensing [30]. As an additional advantage, exploiting just general sensing layers provides cost advantages to sensor fabrication and eases manufacturing complexity, favoring the expansion of sensing devices and their applications by simply changing the nanomaterial-based interface as a function of the class of the substances to be tested. As a last consideration, we are witnessing the widest expansion in machine and deep learning approaches, as testified by the massive growth in the number of publications per year on the subjects. Indeed, just 5000 papers about machine learning in 2012 has risen to more than 80,000 in 2022, and just 200 papers on deep learning in 2012 has grown to more than 65,000 in 2022. Such an explosion in automated learning approaches strongly requires the support of easy-to-produce, versatile measurement devices. The herein proposed non-specific nanogels provide a sound reply, enabling the development of MathMaterial devices suitable for matching with deep and machine learning approaches.

## Data Availability

Raw data supporting the conclusions of this article will be made available by authors upon request with undue reserve.

## Supplementary Materials

The following supporting information can be downloaded at: https://www.mdpi.com/article/10.3390/s222410006/s1, Figure S1: Size distribution of the nanogels, Figure S2: Zeta potential at pH 7.4 of the nanogels, Figure S3: Optical spectra of the surrounding media collected at different times, Figure S4: Image of the device. Table S1: Composition of the nanogels. Table S2: Zeta potential and Z-average of the nanogels.
