# Supplementary material for: Non-Specific Responsive Nanogels and Plasmonics to Design MathMaterial Sensing Interfaces: The Case of a Solvent Sensor
Source: Sensors (Basel). 2022 Dec 19;22(24):10006. doi: 10.3390/s222410006 (PMC9787685; doi:10.3390/s222410006)

Supplementary materials

Non-Specific Responsive Nanogels and Plasmonics to DesignMathMaterial Sensing Interfaces. The Case of a Solvent Sensor

Nunzio Cennamo [1], Francesco Arcadio [1], Fiore Capasso [1], Devid Maniglio [2], Luigi Zeni [1] and Alessandra Maria Bossi [3,*]

[1] Department of Engineering, University of Campania Luigi Vanvitelli, Via Roma 29, 81031 Aversa, Italy; nunzio.cennamo@unicampania.it (N.C.); francesco.arcadio@unicampania.it (F.A.); fiore.capasso@unicampania.it (F.C.); luigi.zeni@unicampania.it (L.Z.)
[2] Department of Industrial Engineering, BIOtech Research Center, University of Trento, Via delle Regole 101, Mattarello, 38123 Trento, Italy; devid.maniglio@unitn.it
[3] Department of Biotechnology, University of Verona, Strada Le Grazie 15, 37134 Verona, Italy
* Correspondence: alessandramaria.bossi@univr.it

Section S1. Synthesis of nanogels

Table S1. Composition of the nanogels:

Sample name	Volume (mL)	Composition (moles)
NIP80	10	BIS 80% + TBAm 4% + MAA 8% + Aam 8%

Solutions were bubbled with nitrogen for 20 minutes, to remove oxygen, then the polymerization was started with the addition of 0.1 mL ammonium persulphate (10% w/v) to the final solution volume and of 0.05 mL of TEMED 0.05 to the final volume.

Section S2. Zeta potential and Z-average

The hydrodynamic size distribution, the associated polydispersity index (PDI) and the Zeta-potential of the nanogels was determined by Dynamic Light Scattering (DLS), and results are reported in Table S2. The corresponding graphs are reported in Figure S1 and Figure S2 respectively.

Table S2. Zeta potential and Z-average of the nanogels

Sample	Measure	$Z_{average}$ (nm)	PDI	ζ-potential (mV)
nanogels	1	89.42	0.187	-29.8
	2	89.61	0.183	-28.9
	3	88.63	0.179	-27.3

Figure S1. Size distribution of the nanogels

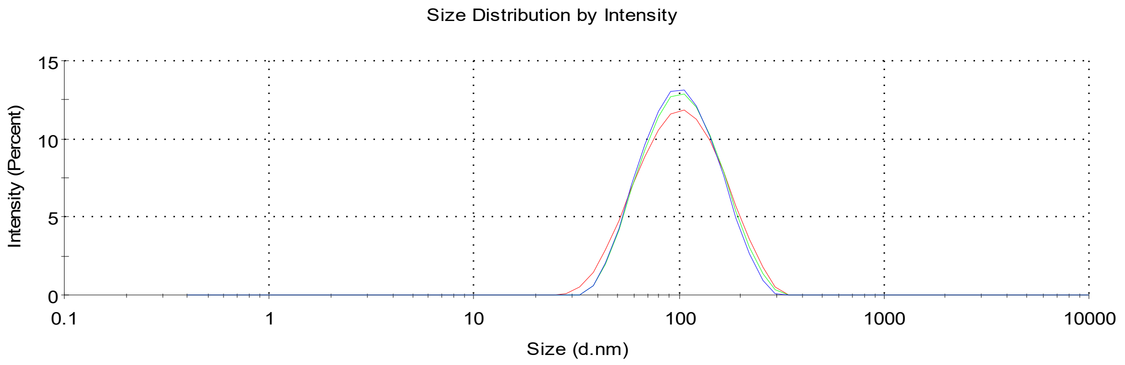

Figure S2. Zeta potential at pH 7.4 of the nanogels

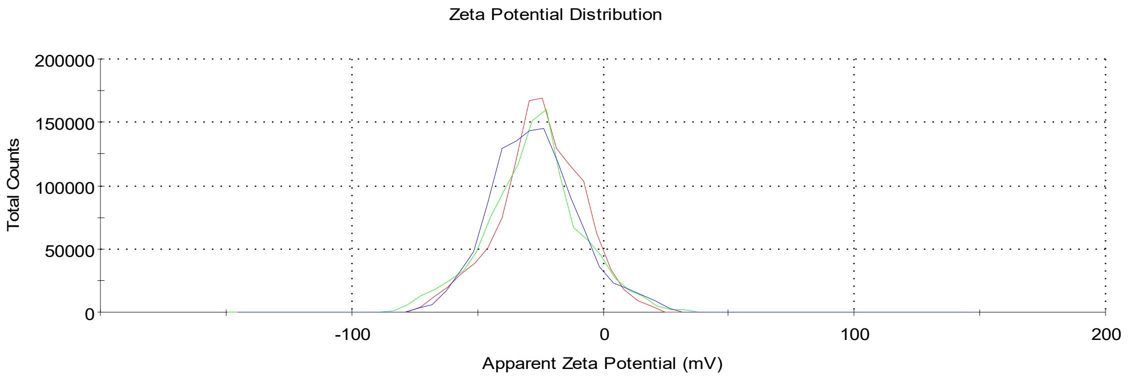

Section S3. Optical spectra collected from the MathMaterial Sensor

Figure S3. Optical spectra of the surrounding media collected at different times.

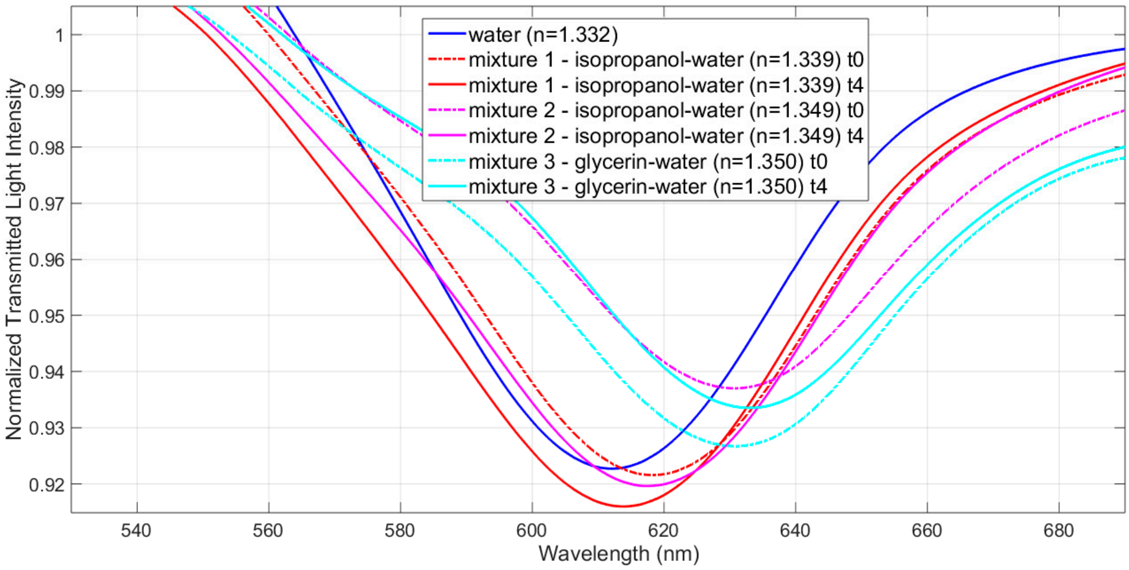

Section S4. Image of the MathMaterial device

Figure S4. Image of the device.

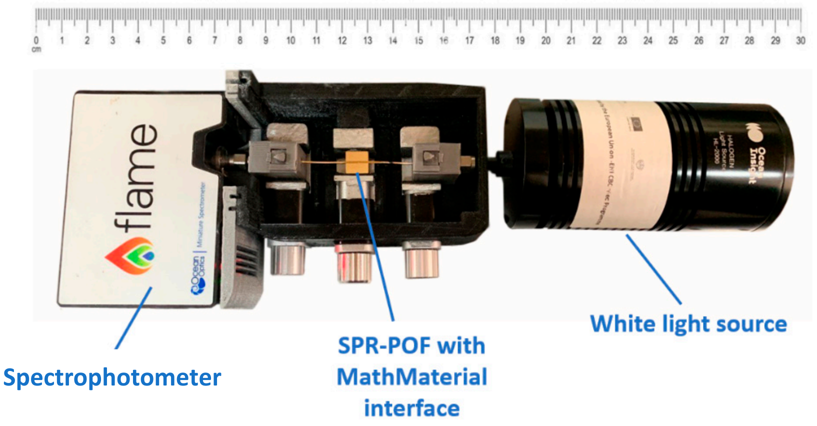

Supplement: Supplementary file 1 [file sensors-22-10006-s001.zip › sensors-2021760-supplementary.pdf]
